# Effect of increasing intraperitoneal infusion rates on bupropion hydrochloride-induced seizures in mice

**DOI:** 10.1186/1744-859X-7-27

**Published:** 2008-12-23

**Authors:** Peter H Silverstone, Robert Williams, Louis McMahon, Rosanna Fleming, Siobhan Fogarty

**Affiliations:** 1 Biovail Corporation, Mississauga, Ontario, Canada; 2 Biovail Technologies, Ltd, Dublin, Ireland; 3 Statistical Department, Biovail Technologies, Ltd, Bridgewater, New Jersey, USA

## Abstract

**Background:**

It is not known if there is a relationship between input rate and incidence of bupropion-induced seizures. This is important, since different controlled release formulations of bupropion release the active drug at different rates.

**Methods:**

We investigated the effect of varying the intraperitoneal infusion rates of bupropion HCl 120 mg/kg, a known convulsive dose_50 _(CD_50_), on the incidence and severity of bupropion-induced convulsions in the Swiss albino mice. A total of 69 mice, approximately 7 weeks of age, and weighing 21.0 to 29.1 g were randomly assigned to bupropion HCl 120 mg/kg treatment by intraperitoneal (IP) administration in 7 groups (9 to 10 animals per group). Bupropion HCl was infused through a surgically implanted IP dosing catheter with infusions in each group of 0 min, 15 min, 30 min, 60 min, 90 min, 120 min, and 240 min. The number, time of onset, duration and the intensity of the convulsions or absence of convulsions were recorded.

**Results:**

The results showed that IP administration of bupropion HCl 120 mg/kg by bolus injection induced convulsions in 6 out of 10 mice (60% of convulsing mice) in group 1. Logistic regression analysis revealed that infusion time was significant (p = 0.0004; odds ratio = 0.974) and increasing the IP infusion time of bupropion HCl 120 mg/kg was associated with a 91% reduced odds of convulsions at infusion times of 15 to 90 min compared to bolus injection. Further increase in infusion time resulted in further reduction in the odds of convulsions to 99.8% reduction at 240 min.

**Conclusion:**

In conclusion, the demonstration of an inverse relationship between infusion time of a fixed and convulsive dose of bupropion and the risk of convulsions in a prospective study is novel.

## Introduction

Bupropion hydrochloride (HCl), a well known antidepressant of the aminoketone class, is associated with a dose-dependent risk of seizures even at therapeutic doses [1-5]. A seizure incidence of 0.4% has been reported for the immediate-release (IR) formulation of bupropion HCl in patients treated with doses in the range 300 to 450 mg/day [3,5]. Subsequently, a seizure incidence of 0.1% was reported for the sustained-release (SR) formulation of bupropion HCl in patients treated with doses of 100 to 300 mg/day [4,6]. In addition, a comparative study of chronic dosing with Wellbutrin SR tablets 150 mg twice daily vs Wellbutrin IR tablets 100 mg three times daily revealed that both formulations were bioequivalent with respect to the rate and extent of absorption but the peak plasma concentrations of bupropion (C_max_) at steady state for the SR tablets were 15% less and T_max_, the time to peak plasma concentrations of bupropion was prolonged than those obtained for the IR formulation [6]. It is not precisely known whether the lower 0.1% seizure incidence observed for the SR formulation of bupropion is due to the latter differences in the same dose of the formulations or the lower dose administered in the SR prospective safety surveillance study [4]. Furthermore, the relationship of different rates of input of bupropion, with the dose kept constant, to the incidence of bupropion-induced seizures remains unknown. Therefore, the objective of this study was to investigate the effect of varying infusion rates of bupropion HCl 120 mg/kg administered intraperitoneally, a dose that has been previously shown to be the convulsive dose_50 _(CD_50_), the dose that will induce convulsions in 50% of mice [7], on the incidence and severity of bupropion-induced convulsions in Swiss albino mice.

## Materials and methods

The study protocol and any amendment(s) or procedures involving the care and use of animals were reviewed and approved by Charles River Laboratories Preclinical Services Inc.'s (CRM) Institutional Animal Care and Use Committee. During the study, the animals were maintained in a facility fully accredited by the Standards Council of Canada (SCC) and the care and use of the animals was conducted in accordance with the guidelines of the Canadian Council on Animal Care (CCAC).

### Animals

A total of 69 female Swiss Crl: CD1 (ICR) albino mice (*Mus Musculus*; Charles River Canada Inc., St Constant, Quebec, Canada) of approximately 7 weeks of age, and weighing 21.0 to 29.1 g were housed individually in stainless steel wire mesh-bottomed cages equipped with an automatic watering valve in an environmentally controlled vivarium (temperature 22 ± 3°C; relative humidity 50 ± 20%) with a 12-h light/dark cycle. Each animal was uniquely identified using an indelible marker and each cage was clearly labelled with a colour-coded cage card indicating group, animal number and sex. All animals were acclimated to their cages and to the light/dark cycle for a period of 7 days prior to surgery and for at least 1 additional day post surgery prior to dosing. In addition, all animals had free access *ad libitum *to a standard certified pelleted commercial laboratory diet (PMI Certified Rodent Diet 5002; PMI Nutrition International Inc., St Louis, MO, USA) and tap water except during designated procedures. Prior to surgery and the initiation of treatment, animals were randomly assigned to 7 treatment groups of 10 mice per group, using a computer-based randomisation procedure that ensures stratification by body weights as follows: group 1, bupropion HCl 120 mg/kg by bolus intraperitoneal (IP) injection; group 2, bupropion HCl 120 mg/kg by IP infusion over 15 min; group 3, bupropion HCl 120 mg/kg by IP infusion over 30 min; group 4, bupropion HCl 120 mg/kg by IP infusion over 60 min; group 5, bupropion HCl 120 mg/kg by IP infusion over 90 min; group 6, bupropion HCl 120 mg/kg by IP infusion over 120 min; and group 7, bupropion HCl 120 mg/kg by IP infusion over 240 min. Animals could be replaced after surgery but prior to the initiation of randomised treatment, due to complications of surgery, illness or death. Additionally, animals in poor health or at the extremes of the prespecified body weight range (18 to 30 g) were not assigned to treatment groups and unassigned animals were released from the study.

### Surgical procedure

Animals assigned to groups 2 to 7 underwent surgery for implantation of an intraperitoneal dosing catheter. Each animal was prepared for surgery by shaving its ventral surface, from the pubis to the thorax, and the interscapular region. The shaved areas of skin were cleaned and disinfected with chlorhexidine gluconate 4% solution, followed by sterile water and then povidone iodine 10% solution. The animals then received a subcutaneous injection of an analgesic/anti-inflammatory agent, caprofen 5 mg/kg, and were anaesthetised using isoflurane anaesthesia during the entire surgical procedure. A bland ophthalmic agent was administered to each eye prior to surgery and was repeated during the procedure as deemed necessary. An approximately 1 cm length of the end of a silicone catheter (internal diameter 0.020 inches, outside diameter 0.037 inches; Medique Products, St Louis, MO, USA) was placed in the peritoneal cavity through a small abdominal skin incision followed by another small incision into the abdomen through the linea alba. The catheter tip was positioned within the abdomen, the catheter secured in place, and the surgical wound closed using non-absorbable suture material. The other end of the catheter was routed subcutaneously through an incision in the interscapular region. Following surgery, the animals were placed in a recovery box with a heat lamp for a few minutes to facilitate recovery, after which they were returned to their home cage, equipped with a flat metal bottom and nesting material.

### Drugs

Bupropion HCl was obtained from Biovail Corporation, Steinbach, Manitoba, Canada, in white powder form, with 100.3% purity, lot number RM0400 and expiry date of October 2007. Vehicle was 0.9% sodium chloride (NaCl) for injection USP and was obtained from Baxter Healthcare Corporation (Deerfield, IL, USA) in clear liquid form, with lot number W6J12C2, and expiry date of January 2008.

The dose formulations of bupropion HCl were prepared on each day. The appropriate amount of bupropion HCl was weighed and dissolved in an appropriate amount of 0.9% NaCl in a suitable container and then vortexed until complete dissolution of the material. On each day of treatment, bupropion HCl 120 mg/kg was administered by IP injection in a dose volume of 10 ml/kg and dose concentration of 12 mg/ml as a bolus (for group 1) or intraperitoneally infused at a dose rate of 40, 20, 10, 6.67, 5 and 2.5 ml/kg/h for groups 2, 3, 4, 5, 6, and 7, respectively. The actual dose administered was based on the most recent body weight of each animal. The dose formulations were kept at room temperature and protected from light.

### Study procedure

All animals were examined twice daily for mortality and signs of ill health or reaction to treatment, except on the days of arrival and necropsy when they were examined only once. After the acclimation period and randomisation, on the day prior to the initiation of treatment, all animals were weighed and the individual body weights were used for dose volume calculation. Treatment was then initiated and lasted for 3 consecutive days with equal numbers of animals from each group dosed on each day. During treatment, all animals were observed continuously for the occurrence of convulsions and for a period of 2 h post completion of infusion of treatment. A 5-min observation period was also conducted at 24 h post dose. Animals were placed in clear Perspex observation boxes during the observation periods. During the observation periods, the number, time of onset, duration and the intensity of the convulsions were recorded. The intensity of each convulsion was graded using Charles River Laboratories, Inc.'s grading system of mild: head and tail slightly extended and little jerking; moderate: head and tail fully extended and some jerking; or severe: head and tail fully extended and strong jerking. In addition, the presence or absence of ataxic gait, paralysis, and catatonic episodes (without a grading of the intensity or number) were recorded over each 15 min observation period. Any animal that had a single episode of severe seizure lasting longer than 1 min or any animal displaying greater than 40 separate episodes of severe convulsions over a 1-h period was killed for humane reasons. At the end of the study, all animals were killed using humane methods.

### Assessment of convulsant activity

The primary outcome variable was the percentage (%) of convulsing mice following treatment. This was the number of animals with convulsions divided by the total number of animals in each group multiplied by 100. The secondary outcome variables were the time to onset of convulsions, mean ± SD convulsions per mouse in each group, the duration of convulsions, and the intensity of convulsions.

### Statistical analysis

Data was summarised and presented in tables by treatment groups for the primary outcome variable, the percentage of convulsing mice, and the four secondary outcome variables, the time to onset of convulsions, mean ± SD convulsions per mouse in each group, duration of convulsions, and intensity of convulsions. For the primary outcome variable, because convulsion, the dependent variable, is a binary outcome, the relationship between the probability of having a convulsion and the infusion rate was analysed using a logistic regression model. The actual infusion times from the seven treatment groups were used as the predictor variable in the logistic regression model. Time to onset of convulsions was analysed using the Cox proportional hazards model to compare each treatment group (groups 2 to 7) and the bolus injection treatment group (group 1). The precise time to onset of convulsions was not recorded for mouse 6 in group 2 due to technical difficulties. Imputation for this missing value was by the mid point of the (known) 15-min observation interval when convulsions started. All mice that did not have convulsions during treatment infusion and by the end of the 2-h post-treatment observation period were considered to be censored at 120 min. A p value of ≤ 0.05 was considered statistically significant.

## Results

In each group 10 animals were dosed, except group 5 where only 9 animals were dosed due to logistical reasons. Following bolus IP injection of bupropion HCl 120 mg/kg to animals in group 1 and intraperitoneal infusion of the same dose of bupropion HCl to animals in groups 3 to 7, no deaths occurred. However, in group 2 (15 min infusion time), two animals were killed *in extremis *for humane reasons within 15 and 75 min of dosing, respectively.

Following administration of treatment, a variety of clinical signs was observed in mice across all treatment groups including ataxic gait, catatonia, decreased activity and/or paralysis. The number of animals with the observed clinical signs varied inversely with the infusion time, with the animals administered IP bupropion HCl 120 mg/kg by bolus injection showing the most number of clinical signs. Paralysis and/or lying on the side were observed in 8 out of 10 mice in group 1.

### Percentage of convulsing mice

The IP administration of bupropion HCl 120 mg/kg by bolus injection induced convulsions in 6 out of 10 mice (60% of convulsing mice) in group 1. Increasing the total time of IP infusion of bupropion HCl to 15 min (group 2) while maintaining the dose constant (120 mg/kg) was associated with an increase in the percentage of convulsing mice to 90%. Subsequent increases in the IP infusion time of bupropion HCl 120 mg/kg to 30, 60, 90, 120 and 240 min were associated, in general, with a corresponding decrease in the percentage of convulsing mice to 50%, 50%, 0%, 20% and 0%, respectively, for mice in groups 3 to 7 (Table 1).

**Table 1:** Effect of bupropion HCl 120 mg/kg intraperitoneal infusion times on bupropion HCl-induced percentage of convulsing mice and on odds of convulsing vs not convulsing

Group*	**Intraperitoneal (IP) infusion time (min)**	**No. of mice convulsing**	**Convulsing mice**	**Reduction in odds of convulsing†**
1	0 (bolus)	6	60%	-
2	15	9	90%	32.6%
3	30	5	50%	54.6%
4	60	5	50%	79.4%
5	90	0	0%	90.6%
6	120	2	20%	95.8%
7	240	0	0%	99.8%

*Number of mice was 10 for each group, except group 5 that had 9 mice.

†Reduced odds of convulsing vs not convulsing for any infusion time, x min, were calculated using the formula: 1 - (0.974^x^) × 100%; where 0.974 was the odds ratio estimate obtained from logistic regression analysis, and x = infusion time (min).

Logistic regression analysis of treatment infusion time vs the probability of having a convulsion showed that infusion time was statistically significant (p = 0.0004) with an estimated odds ratio of 0.974, indicating that when infusion time increases by 1 min, the odds (or probability) for a mouse having a convulsion vs not having a convulsion decreases by 2.6%. Using the estimated odds ratio, the decreases in the probability for a mouse having a convulsion were calculated for the infusion times used in groups 2 to 7 (Table 1). The results show that as infusion time increases, with dose kept constant, mice are at progressively reduced odds of having convulsions, with the odds markedly reduced by 55% with the infusion time of 30 min, by 91% with infusion time of 90 min, and almost zero at 240 min. These results also confirm that the initial increase in the percentage of convulsing mice observed at the IP infusion time of 15 min is random.

### Time to onset of convulsions

The time to onset of convulsions for the individual mouse in each group are shown in Table 2. The observed shortest times to onset of first convulsions were 5, 6 and 7 min in group 1, the bolus injection group, while the observed longest times to onset of first convulsions were 70, 69 and 60 min in group 4, the 60 min infusion time group. Between groups 1 and 4, there was a progressive increase in the time to onset of first convulsions with increase in infusion time. The results of the Cox proportional hazards model showed that infusion time was statistically significant (p = 0.0002) with a hazard ratio of 0.978, indicating a 2.2% reduction in the hazard (probability) of a mouse having a convulsion when the infusion rate increases by 1 min. This hazard ratio is consistent with the odds ratio obtained by logistic regression analysis above.

**Table 2:** Effect of bupropion HCl 120 mg/kg intraperitoneal infusion times on time to onset of bupropion HCl-induced convulsions and on odds of convulsing vs not convulsing in mice

Group*	**Intraperitoneal (IP) infusion time (min)**	**Time to Onset of Convulsions for Individual Mice (min)†**	**Reduction in hazard (probability) of convulsing**
1	0 (bolus)	7; 30; 6; 120; 5; 11; 120; 120; 23; 120	-
2	15	15; 120; 5; 18; 19; 23; 14; 20; 16; 13	28.4%
3	30	20; 120; 17; 120; 120; 120; 120; 39; 42; 31	48.7%
4	60	32; 120; 42; 120; 120; 60; 120; 70; 120; 69	73.7%
5	90	120; 120; 120; 120; 120; 120; 120; 120; 120	86.5%
6	120	120; 9; 41; 120; 120; 120; 120; 120; 120; 120	93.1%
7	240	120; 120; 120; 120; 120; 120; 120; 120; 120; 120	99.5%

*Number of mice was 10 for each group, except group 5 that had 9 mice.

†Mice that did not have convulsions during treatment infusion and by the end of the 2-h post-treatment period were considered to be censored at 120 min.

‡Reduced hazard of convulsing vs not convulsing for any infusion time, x min, was calculated using the formula: 1 - (0.978^x^) × 100%; where 0.978 was the hazard ratio estimate obtained from Cox proportional hazards model, and x = infusion time (min).

### Mean ± SD convulsions per mouse

The IP administration of bupropion HCl 120 mg/kg by bolus injection (group 1) induced a total of 155 episodes of convulsions or 15.5 ± 22.4 mean ± SD convulsions per mouse. Increasing the IP infusion time for the same dose of bupropion HCl to 15 min (group 2) resulted in an increase in the total number of convulsions to 272 convulsions or 27.2 ± 39.5 mean ± SD convulsions per mouse (Table 3). The odds ratio and the hazard ratio results indicate that this increase is random. Further increase in IP infusion times to 30 and 60 min (groups 3 and 4) resulted in 161 or 16.1 ± 22.2 and 170 or 17.0 ± 22.8 total or mean ± SD convulsions per mouse, respectively, which were similar to the results obtained for the bolus injection (group 1). Additional increase in IP infusion times for the same dose of bupropion HCl to 90, 120 and 240 min, resulted in either no convulsions or a minimal number of total or mean convulsions per mouse (Table 3). Overall, IP infusion times of 0 to 60 min generally resulted in a stable number of mean convulsions per mouse, while increases in infusion time to ≥ 90 min resulted in either no convulsions or a minimal number of mean convulsions per mouse.

**Table 3:** Effect of bupropion HCl 120 mg/kg intraperitoneal infusion times on the mean ± SD bupropion HCl-induced convulsions per mouse

Group*	**Intraperitoneal (IP) infusion time (min)**	**Total no. of convulsions**	**Mean ± SD convulsions per mouse**
1	0 (bolus)	155	15.5 ± 22.4
2	15	272	27.2 ± 39.5
3	30	161	16.1 ± 22.2
4	60	170	17.0 ± 22.8
5	90	0	0
6	120	34	3.4 ± 10.4
7	240	0	0

*Number of mice was 10 for each group, except group 5 that had 9 mice.

### Duration and intensity of convulsions

The mean numbers of short, medium and long duration convulsions were generally stable for infusion times of 0 to 60 min (Tables 4 and 5). Infusion times of ≥ 90 min resulted in either no convulsions or a minimal number of short, medium and long convulsions. Within the groups that had convulsing mice, for each IP infusion time, the mean ± SD numbers of short duration convulsions were highest followed by the medium and then the long convulsions. Overall, the trend of the results obtained for the intensity of convulsions was similar to that observed for the duration of convulsions except in group 1 where there were no severe convulsions (Tables 4 and 5).

**Table 4:** Effect of bupropion HCl 120 mg/kg intraperitoneal infusion times on the mean ± SD duration of bupropion HCl-induced convulsions in mice

Group*	**Intraperitoneal (IP) infusion time (min)**	**Mean ± SD duration of convulsions**		
		**Short** **(0 to 10 s)**	**Medium** **(11 to 30 s)**	**Long** **(= 31 s)**
1	0 (bolus)	11.2 ± 15.6	2.6 ± 3.9	1.6 ± 4.4
2	15	19.9 ± 31.4	4.8 ± 8.0	2.3 ± 3.3
3	30	12.2 ± 15.7	2.5 ± 4.1	1.4 ± 4.1
4	60	12.0 ± 17.7	3.8 ± 7.1	1.0 ± 1.7
5	90	0	0	0
6	120	3.1 ± 9.5	0.2 ± 0.6	0.1 ± 0.3
7	240	0	0	0

*Number of mice was 10 for each group, except group 5 that had 9 mice.

**Table 5:** Effect of bupropion HCl 120 mg/kg intraperitoneal infusion times on the mean ± SD intensity of bupropion HCl-induced convulsions in mice

Group*	**Intraperitoneal (IP) infusion time (min)**	**Mean ± SD intensity of convulsions**		
		
		**Mild**	**Moderate**	**Severe**
1	0 (bolus)	10.0 ± 12.9	5.5 ± 10.4	0
2	15	14.2 ± 17.4	7.1 ± 10.9	5.9 ± 13.1
3	30	9.2 ± 11.7	4.1 ± 6.4	2.8 ± 8.9
4	60	10.2 ± 16.4	4.6 ± 7.0	2.2 ± 4.8
5	90	0	0	0
6	120	2.8 ± 8.5	0.5 ± 1.6	0.1 ± 0.3
7	240	0	0	0

*Number of mice was 10 for each group, except group 5 that had 9 mice.

## Discussion

When used as an antidepressant, to minimise the risk of seizures, the labels for the various formulations of bupropion HCl recommend: (i) that the total daily dose of Wellbutrin and Wellbutrin XL not exceed 450 mg/day, and Wellbutrin SR not exceed 400 mg/day; (ii) a maximum single dose and minimum time interval between single doses to avoid high peak concentrations of bupropion and/or its metabolites; and (iii) the rate of incrementation of dose be gradual [5,6,8]. It is also recommended that significant caution be exercised when administering bupropion HCl to patients with predisposing factors that may increase the risk of seizures, which include patient factors and the use of concomitant medications. However, these recommendations are based strictly on retrospective analysis of clinical experience gained during the development of bupropion [5,6,8]. There are no published studies of the effect of different rates of administration of a fixed and convulsive dose of bupropion HCl on the incidence and severity of bupropion HCl-induced seizures in animals or humans. Hence, this study was designed to investigate the effect of different infusion rates of a fixed, convulsive dose of bupropion HCl, previously shown to be a CD_50_, in Swiss albino mice [7].

The results of this study show that IP administration of bupropion HCl 120 mg/kg by bolus injection induced convulsions in 60% of mice and increasing the IP infusion time of this fixed dose was initially associated with an increase in the percentage of convulsing mice at 15 min infusion time followed generally by a decrease to 0% of convulsing mice at 90 min. Logistic regression analysis showed that infusion time was statistically significant and that the strength of the latter association (odds ratio) was less than 1, confirming that the odds of having convulsions decreased with increase in infusion time. The odds ratio also confirmed that the increase in the percentage of convulsing mice at the 15 min infusion time was a random occurrence. Furthermore, using the odds ratio to calculate the reduction in the odds of convulsions for each infusion time used in the study (15, 30, 60, 90, 120 and 240 min) revealed there was a marked reduction (91%) in the risk of convulsions during the first 90 min of infusion time, after which the risk of convulsions continued to decrease to an almost complete reduction (99.8%) at 240 min. The magnitude of the hazard ratio, an estimate of the relative risk, obtained from the Cox proportional hazards model used to analyse the time to onset of convulsions was consistent with the odds ratio, indicating the robustness of the relationship between increasing infusion time and reduction in the risk of convulsions.

A consistent trend was observed in the results of the other secondary outcome variables, the mean convulsions per mouse, duration of convulsions, and intensity of convulsions. Overall, infusion times of 15 to 60 min (groups 2 to 4) were associated with stable mean convulsions per mouse, a stable number of mean short, medium and long convulsions, as well as a stable number of mild, moderate and severe convulsions compared to the bolus injection group (group 1); however, there were no severe convulsions in group 1. Infusion times of ≥ 90 min resulted in either no convulsions (at 90 and 240 min) or a minimal number of convulsions. In addition, the number of animals observed with clinical signs following treatment was higher in mice in the bolus injection group and then decreased with increase in infusion time. The reason why the results of these secondary outcome variables were stable for infusion times of 0 to 60 min while there was a marked and progressive reduction in the odds of convulsions during the same infusion time range is not known. Additionally, the precise aetiology of bupropion-induced seizures is unknown, and it is not known whether the risk is due principally to the parent drug or to one of its three major metabolites or a combination of more than one of the four [2,9,10]. However, it is well established that bupropion HCl-induced convulsions are dose dependent, and hence, concentration dependent [1-6,9]. The C_max _of a drug is determined by the dose, rate of input and elimination rate. Since the dose was kept constant in this study, and assuming that the elimination rate was constant as well during the study, the C_max _of bupropion would be determined by the rate of input of bupropion HCl. Therefore, it may be hypothesised that for infusion times of between 0 to 60 min, there was a progressive reduction in the risk of convulsions and hence, the observed number of mice with convulsions. However, in those mice that had convulsions, the concentrations of blood bupropion and/or the metabolites were high enough to induce convulsions of the same frequency (mean convulsions per mouse), duration and intensity. At infusion times of ≥ 90 min, however, the combined effect of the reduction in the risk of convulsions and the lower concentrations of blood bupropion and/or the metabolites resulted in a minimal number of convulsions or did not induce any convulsions at all. The findings that the time to onset of convulsions progressively increased and the number of animals with observed clinical signs progressively decreased with increase in infusion time from 0 to 60 min supports this 'threshold' hypothesis.

The IP route of bupropion HCl administration was used in this study because it is considered to be a route that involves the first-pass effect and, therefore, gains access to the hepatic portal system, making it more similar to oral administration [11]. The high dose of bupropion HCl used in this study was to ensure that convulsions occurred in the mice, and it is noteworthy that even higher doses have been recorded in overdose experience in humans previously. The label for Wellbutrin XL indicates that overdoses of up to 17,500 mg (equals 250 mg/kg for 70 kg adult) of the immediate-release formulation have been reported in humans since the introduction of bupropion and seizure was reported only in one-third of all cases [8]. The time to onset of first convulsion of 5 min observed in this study is consistent with previous reports of median latency to first convulsions obtained in male Swiss albino mice of 6 (3.5 to 8.15) and 6.71 (4.83 to 7.16) min following the IP administration of an anticonvulsant dose of bupropion HCl 160 and 75 to 150 mg/kg [7,12], respectively. The latter finding confirms the validity of this epiletogenic experimental model.

The bupropion HCl used in this study was the powder dissolved in saline, which is very similar to the immediate-release formulation used clinically. The immediate-release formulation of bupropion HCl has been previously reported to have a T_max _of 15.6, 23.4 and 15.6 min for bupropion parent drug, hydroxybupropion (BW 306U) and threohydrobupropion (BW A494U), respectively, following IP administration of bupropion HCl 40 mg/kg (Suckow *et al*.) [11].

In conclusion, we have demonstrated that increasing the IP infusion time of a fixed dose of bupropion HCl 120 mg/kg, a CD_50 _in mice, was associated with a 91% markedly reduced odds of convulsions at infusion times of 15 to 90 min compared to bolus injection. Further increase in infusion time resulted in further reduction in the odds of convulsions to 99.8% reduction at 240 min. The hazard ratio obtained from the analysis of time to onset of convulsions using the Cox proportional hazards model was consistent with the odds ratio, confirming the robustness of the strength of the relationship between infusion time and the risk of convulsions. In contrast, generally a similar number of mean convulsions per mouse, duration and intensity of convulsions were observed at infusion times of 15 to 60 min compared to bolus injection. Infusion times of ≥ 90 min resulted in either a minimal number of convulsions or no convulsions at all. The demonstration of an inverse relationship between infusion time of a fixed and convulsive dose of bupropion and the risk of convulsions is novel.

## Competing interests

The authors declare that they have no competing interests.

## Authors' contributions

PHS participated in the design of the study and drafted the manuscript. RW participated in the design of the study and its coordination. LM participated in the design of the study and its coordination. RF performed the statistical analysis. SF participated in the design of the study and its coordination. All authors read and approved the final manuscript.
